# Bisphenol a Disrupts Steroidogenesis and Induces Apoptosis in Human Granulosa Cells Cultured In Vitro

**DOI:** 10.3390/ijms26094081

**Published:** 2025-04-25

**Authors:** Dominika Celar Šturm, Tadeja Režen, Nina Jančar, Irma Virant-Klun

**Affiliations:** 1 Clinical Research Centre, University Medical Centre Ljubljana, 1000 Ljubljana, Slovenia; irma.virant@kclj.si; 2 Centre for Functional Genomics and Bio-Chips, Institute of Biochemistry and Molecular Genetics, Faculty of Medicine, University of Ljubljana, 1000 Ljubljana, Slovenia; tadeja.rezen@mf.uni-lj.si; 3 Department of Human Reproduction, Division of Obstetrics and Gynaecology, University Medical Centre Ljubljana, 1000 Ljubljana, Slovenia; nina.jancar@kclj.si

**Keywords:** bisphenol A, human granulosa cells, steroidogenesis, apoptosis, steroid hormones

## Abstract

Bisphenol A (BPA) is a common synthetic chemical compound classified as an endocrine disruptor. It affects multiple physiological systems in the body, including the female reproductive system, particularly granulosa cells (GCs) in the ovaries, where steroidogenesis occurs. This study investigated the impact of various BPA concentrations (environmentally relevant concentrations of 0.001 µM and 0.1 µM and toxicological concentration of 100 µM) and exposure times (24 and 72 h) on cell viability and counts and in vitro production of estradiol and progesterone in human GCs collected from waste follicular fluid of IVF patients. Gene expression analysis of 182 genes associated with steroidogenesis and apoptosis was performed in GCs using PCR arrays, followed by protein expression analysis by Western blot. Our results demonstrate that after longer BPA exposure (72 h), a higher concentration of BPA (100 µM) negatively affects the cellular viability and counts and significantly alters steroid hormone biosynthesis in vitro, leading to reduced concentrations of estradiol and progesterone in the culture medium. We found that all BPA concentrations altered the expression of different steroidogenesis- and apoptosis-related genes in GCs. At 0.001 μM, BPA exposure decreased the expression of *TRIM25*, *UGT2B15*, *CASP3*, and *RPS6KA3* genes and increased the expression of *NR6A1* and *PPID* genes. At 0.1 μM, BPA increased the expression of *AR*, *HSD3B1*, *BID*, *IKBKG*, and *PPID* genes while reducing the expression of *TRIM25* and *CASP3* genes. At the highest concentration of 100 μM, BPA upregulated the expression of *AR*, *GPER30*, *BID*, *IKBKG*, and *PPID* genes and downregulated the expression of *FOXO1* and *UGT2B15* genes. These results highlight BPA’s concentration-specific effects on steroidogenesis and apoptosis and show its potential to compromise GC function, with possible negative implications for female fertility and ovarian health, even at environmentally relevant concentrations.

## 1. Introduction

Bisphenol A (BPA) is a synthetic chemical compound commonly used to manufacture plastics, epoxy resins, food packaging, and clothing [1]. Due to its widespread global presence, it has become, and persists as, a significant health concern. It is estimated that oral consumption through food ingestion contributes to more than 90% of all BPA exposure, while other sources of BPA contamination only add up to about 5% of exposure through direct skin contact or inhalation [2,3]. BPA is classified as an endocrine-disrupting chemical (EDC), as it can interfere with an organism’s hormonal system, having the ability to mimic or antagonize the activity of the natural hormone estrogen, thus disrupting the hormonal homeostasis in the body [4]. One of the most sensitive targets of BPA’s harmful effects is the female reproductive system, particularly the follicles and follicular cells, including granulosa cells (GCs), where steroidogenesis occurs [4].

BPA mainly exerts its harmful effects on reproductive health through its interactions with multiple cellular receptors, including estrogen receptors (ERα and ERβ), androgen receptors (ARs), and membrane-bound receptors such as the G protein-coupled estrogen receptor (GPER) [5]. These interactions affect many biological processes by modulating important signaling pathways and can lead to altered gene expression and impaired cellular functions. Beyond the classical steroid hormone receptors, BPA can interact with other nuclear receptors such as the pregnane X receptor (PXR) and peroxisome proliferator-activated receptor gamma (PPARγ), which play roles in metabolism and cellular homeostasis. BPA’s broad receptor-binding capability highlights its potential to interfere with multiple signaling systems, contributing to a range of pathologies, including infertility, premature ovarian insufficiency (POI), polycystic ovary syndrome (PCOS), and other reproductive and metabolic disorders [6,7].

GCs are somatic follicular cells, essential for regulating the production of sex steroid hormones and ensuring normal oocyte maturation and folliculogenesis. They play a critical role in the synthesis of steroid hormones, such as estradiol and progesterone, which are crucial for ovulation, corpus luteum formation, and the maintenance of pregnancy. Steroid hormones are produced through complex steroidogenic pathways, regulated by enzymes and transcriptional factors such as StAR, CYP11A1, HSD3B1, CYP19A1, etc., ensuring proper cholesterol conversion into biologically active steroids. Numerous studies using human and animal models support this possibility, reporting that both toxicologically higher and even lower environmentally relevant doses of BPA, at nano- and micromolar concentrations, can affect the hypothalamus–pituitary–gonadal axis, hormone balance, folliculogenesis, number of ovarian follicles, steroidogenesis-related gene expression, and ovarian apoptosis [5,8,9,10,11].

Moreover, BPA’s impact on GCs extends even further than steroidogenesis. BPA has been shown to induce oxidative stress and the production of reactive oxygen species (ROS) and disturb cellular antioxidant defense mechanisms [12]. Elevated ROS can lead to mitochondrial dysfunction and trigger the intrinsic apoptotic pathway, contributing to poorer GC survival, impairing follicular development and oocyte quality, leading to elevated follicular atresia. Granulosa cell apoptosis can also lead to premature ovarian insufficiency, ultimately reducing female fertility [13].

The selected environmentally relevant concentrations of BPA in this study were derived from the findings of more studies that measured BPA levels in the follicular fluid of women undergoing in vitro fertilization (IVF) procedures. The mean BPA concentrations reported in these studies range from 0.000467 µM to 0.0937 µM [14,15,16,17,18], with some individual maximum measured concentrations being significantly higher (e.g., 0.357 µM, 0.651 µM [17,18]). Therefore, the selected environmentally relevant concentrations include both lower and higher reported BPA levels in follicular fluid and allow for comparison with the actual concentrations detected in follicular fluid of women from different parts of the world.

In addition to environmentally relevant concentrations, we included a high dose of BPA, 100 µM, to assess potential toxicological effects and explore cellular responses that may not show at lower exposures. Using this toxicological concentration allowed us to examine mechanistic pathways and identify possible non-monotonic dose–response relationships.

The aim of this study was to investigate if and how BPA at environmentally relevant (0.001 µM and 0.1 µM) and toxicological (100 µM) doses at two different time exposures (24 and 72 h) affects steroidogenesis and apoptosis, based on the expression levels of 182 genes involved in steroidogenesis and apoptosis. Our experimental model was of human GCs, obtained and isolated from waste follicular fluid after oocyte retrieval from young patients of reproductive age, who were partners of infertile men undergoing an in vitro fertilization procedure for infertility treatment.

## 2. Results

Our data show that exposure to different BPA concentrations affects GCs in different ways.

### 2.1. Cell Counts and Viability After BPA Exposure

The results show that BPA exposure affects GC viability in a time- and concentration-dependent manner.

**Figure 1:**
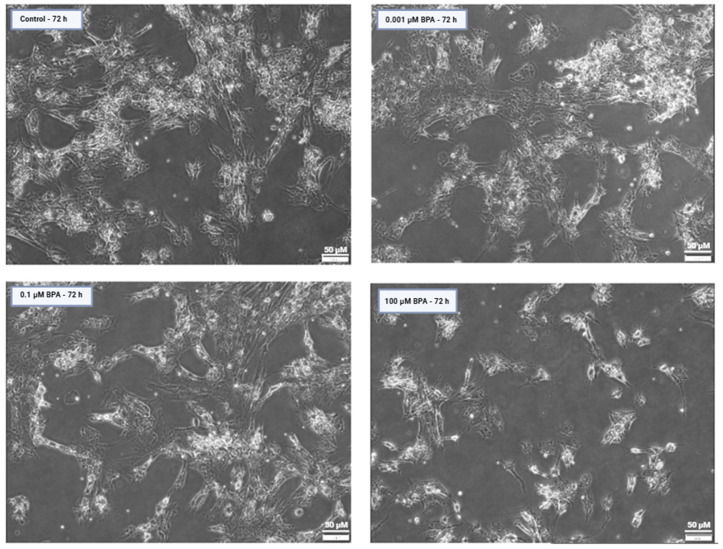
Phase-contrast images of human granulosa cells at 20× magnification, directly exported from the CellSens app, showing attachment to the bottom of the culture dish after 72 h of incubation in a control group and at different concentrations of BPA. The cells exhibit a mesenchymal-like phenotype, with a noticeable reduction in cell density at 100 µM BPA compared with the other conditions. The images were captured using an Olympus IX73 microscope and the CellSens 3.2. software, (Evident Corporation, Tokyo, Japan). Scale bar: 50 µm.

**Figure 2:**
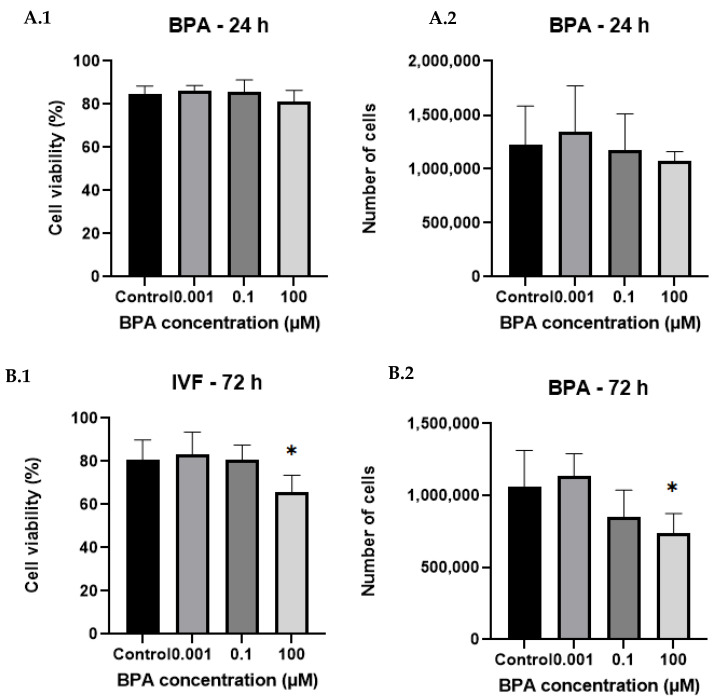
The effects of different BPA concentrations and exposure times on human granulosa cell viability and cell numbers compared with the BPA non-treated control. After 24 h (**A.1**,**A.2**), there were no statistically significant differences in either cell viability or cell number. However, after 72 h of BPA exposure (**B.1**,**B.2**), BPA at a concentration of 100 µM caused a significant decrease in both cell viability and cell count. * Statistically significant (*p* < 0.05).

At 24 h of exposure, BPA exhibited no significant effect on GCs, as there was no observed impact on cell viability or cell count compared with the control, regardless of BPA concentration. On the other hand, BPA significantly reduced the viability and number of GCs after 72 h of BPA exposure and at the highest concentration of 100 µM (*p* < 0.05, Figure 1 and Figure 2). There was also a slight decrease in the GC number observed at the environmentally relevant BPA concentration of 0.1 µM after 72 h, although this was not significant.

### 2.2. In Vitro Progesterone and Estradiol Synthesis: Concentrations in Spent Culture Medium

**Figure 3:**
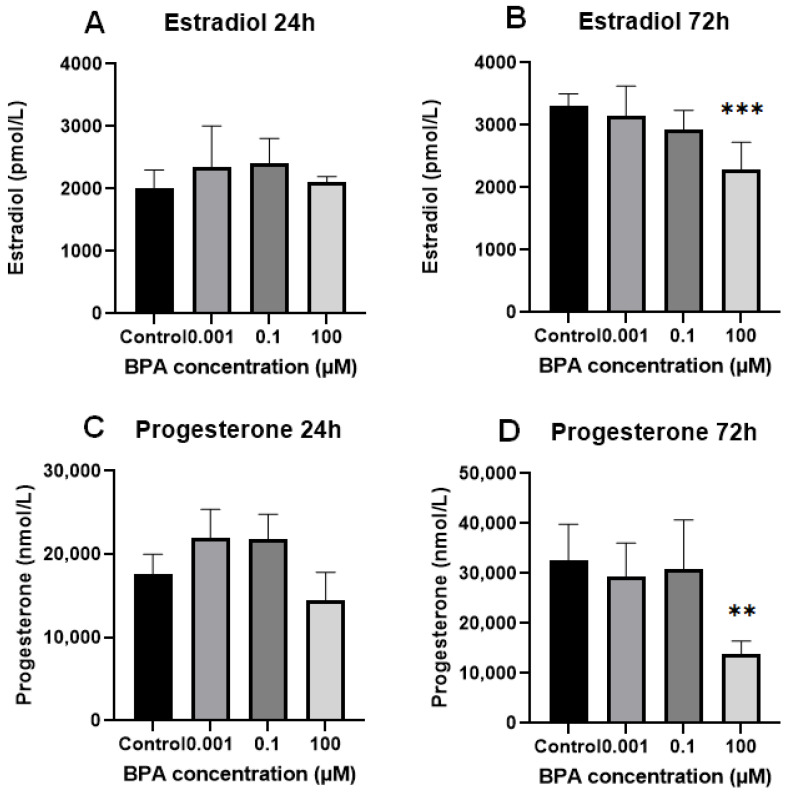
In vitro estradiol and progesterone production assessed using spent culture medium analysis. After 24 h, there was no significant effect of BPA on estradiol (**A**) and progesterone (**C**) biosynthesis in vitro. However, a trend was observed where lower BPA concentrations (0.001 µM, 0.1 µM) appeared to increase the production of both estradiol and progesterone. After 72 h, a concentration of 100 µM BPA significantly reduced the synthesis of both estradiol (**B**) and progesterone (**D**) (*p* = 0.0004 and *p* = 0.0050, respectively). The control values at 72 h were 3304.0 ± 198.4 pmol/L for estradiol (**B**) and 32,508.0 ± 7339.0 nmol/L for progesterone (**D**). ** statistically significant at *p* < 0.01; *** statistically significant at *p* < 0.001.

No significant differences were observed in estradiol and progesterone levels in the spent culture medium between the BPA-treated groups and the GC control group at the 24 h time point. After 72 h of cell exposure to BPA, statistically significant decreases in progesterone and estradiol concentrations were observed at the maximum dose of BPA (100 µM) compared with the control group (Figure 3).

**Figure 4:**
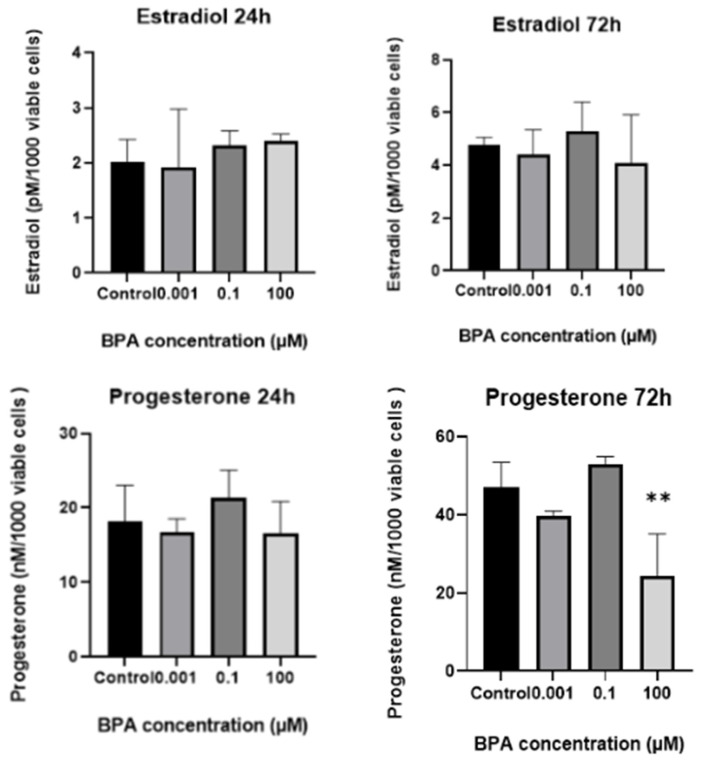
In vitro estradiol and progesterone production assessed using spent culture medium analysis. The bar charts display progesterone and estradiol concentrations, normalized to 1000 viable cells following 24 and 72 h of exposure to BPA concentrations of 0.001 µM, 0.1 µM, and 100 µM. For estradiol, no significant differences were observed among the groups at either 24 or 72 h of BPA exposure. For progesterone, no significant differences were observed among the groups at 24 h; however, after 72 h, there was a significant decrease in progesterone production at 100 µM concentration of BPA compared with the control group (*p* = 0.00632). ** statistically significant (*p* < 0.01).

When comparing groups of cells over time, a significant increase in progesterone concentration in the spent medium was observed only in the GC control group. No significant changes in progesterone levels were detected in the BPA-treated groups over time. The progesterone concentration in the spent culture medium remained almost the same at the 100 µM concentration of BPA (*p* > 0.999), while at 0.001 µM and 0.1 µM BPA, the concentrations increased slightly, though not significantly (*p* = 0.1990 and *p* = 0.0683, respectively). For estradiol, a significant increase in concentration in the spent medium was observed over time in the control group and at the lowest (0.001 µM) concentration of BPA (*p* < 0.0001 and *p* = 0.0061). In contrast, no significant increase in estradiol concentration was noted in the spent medium of GCs exposed to 0.1 µM and 100 µM BPA (*p* = 0.10308 and *p* = 0.8587). When hormone concentrations were adjusted to 1000 viable cells (Figure 4), there were no statistically significant differences in progesterone or estradiol concentrations in the spent culture medium at the 24 h mark between the groups. However, after 72 h, the normalized data showed a similar pattern to the non-normalized data for progesterone production: treatment of cells with a 100 µM concentration of BPA still caused a strong reduction in progesterone levels in the spent culture medium. For estradiol, the normalized data showed no significant differences between groups of cells, with estradiol production remaining unchanged over time.

### 2.3. Differential Expression of Genes Associated with Steroidogenesis and Apoptosis in GCs After BPA Exposure

**Figure 5:**
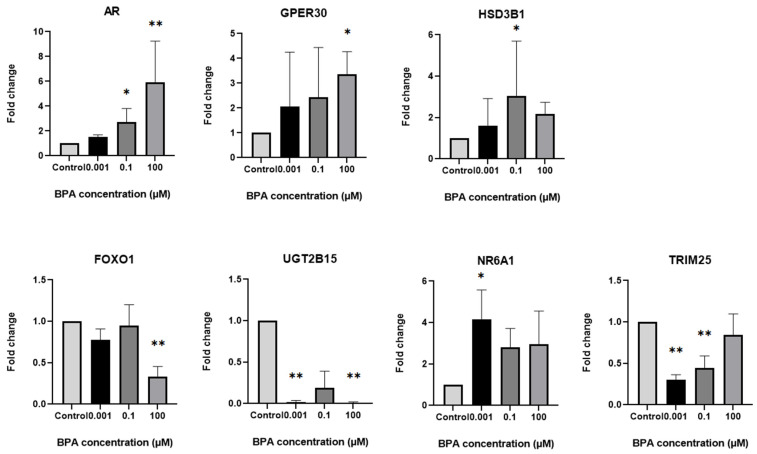
Effects of different concentrations of BPA on the expression of genes associated with steroidogenesis in granulosa cells after 72 h of exposure. Total messenger RNA (mRNA) was extracted, reverse transcribed, and amplified using qPCR. Gene expression was normalized using the geometric mean of three reference genes (*GAPDH*, *HPRT1*, and *GUSB*). The results are expressed as the fold change (2^(−ΔΔCT) ± standard deviation. This figure shows genes with significantly altered expression. At 0.001 µM, BPA significantly changed the expression of *NR6A1* (*p* = 0.0270), *TRIM25* (*p* = 0.0011), and *UGT2B15* (*p* = 0.0030). At 0.1 µM, BPA significantly changed the expression of *AR* (*p* = 0.0400), *HSD3B1* (*p* = 0.0409), and *TRIM25* (*p* = 0.0098). At 100 µM, BPA significantly changed the expression of *AR* (*p* = 0.0016), *GPER30* (*p* = 0.0151), *FOXO1* (*p* = 0.0025), and *UGT2B15* (*p* = 0.0031). * statistically significant at *p* < 0.05, ** statistically significant at *p* < 0.01.

**Figure 6:**
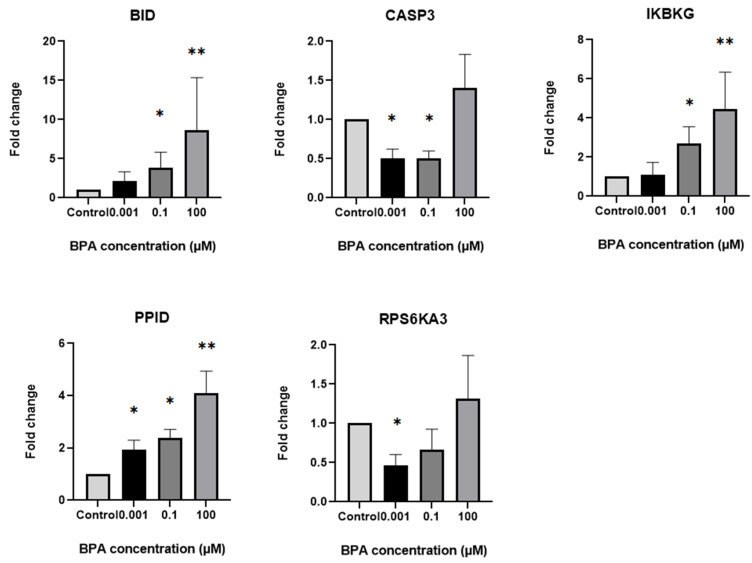
Effects of different concentrations of BPA on the expression of genes associated with apoptosis in granulosa cells after 72 h of exposure. Total messenger RNA (mRNA) was extracted, reverse transcribed, and amplified using qPCR. Gene expression was normalized using the geometric mean of three reference genes (*GAPDH*, *HPRT1*, and *GUSB*). The results are expressed as the fold change (2^(−ΔΔCT) ± standard deviation). This figure shows significantly altered expression of genes after 72 h of incubation. At 0.001 µM, BPA significantly changed the expression of *CASP3* (*p* = 0.0185), *PPID* (*p* = 0.045), and *RPS6KA3* (*p* = 0.0336). At 0.1 µM, BPA changed the expression of *BID* (*p* = 0.0410), *CASP3* (*p* = 0.0134), *IKBKG* (*p* = 0.0412), and *PPID* (*p* = 0.0370), while at 100 µM, BPA changed the expression of *BID* (*p* = 0.0042), *IKBKG* (*p* = 0.0053), and *PPID* (*p* = 0.0062). * statistically significant at *p* < 0.05, ** statistically significant at *p* < 0.01.

Analysis of mRNA expression, conducted using ANOVA with Dunnett’s post hoc test, revealed no statistically significant changes in gene expression in GCs incubated with BPA for 24 h. In contrast, GCs incubated with BPA for 72 h exhibited significant alterations in gene expression, even at lower, environmentally relevant concentrations of BPA. There were 12 genes—seven steroidogenesis-related (*NR6A1*, *TRIM25*, *UGT2B15*, *HSD3B1*, *AR*, *GPER30*, and *FOXO1*) and five apoptosis-related (*PPID*, *CASP3*, *RPS6KA3*, *BID*, and *IKBKG*) genes—that were significantly upregulated or downregulated in GCs following exposure to different concentrations of BPA. Significantly altered expressions of genes and their fold changes are depicted in Figure 5 for steroidogenesis-related genes and Figure 6 for apoptosis-related genes. These results suggest that BPA elicits slow cellular responses after longer BPA exposure time and can modify the expression of numerous apoptosis and steroidogenesis-related genes.

### 2.4. Differential Expression of Steroidogenesis and Apoptosis-Related Genes in GCs Based on Exposure to Different BPA Concentrations

For steroidogenesis-related genes, a 0.001 µM concentration of BPA led to increased expression of *NR6A1* (*p* = 0.0270) and decreased expression of *TRIM25* and *UGT2B15* (*p* = 0.0011 and *p* = 0.0030) (Figure 5). For apoptotic genes, exposure of GCs to this BPA concentration increased the expression of *PPID* (*p* = 0.045) and decreased the expression of *CASP3* and *RPS6KA3* (*p* = 0.0185 and *p* = 0.0336), as shown in Figure 6.

Exposure of GCs to the next-higher concentration of BPA (0.1 µM) upregulated the expression of steroidogenesis-related genes *HSD3B1* and *AR* (*p* = 0.0409 and *p* = 0.0400) and downregulated the expression of steroidogenesis-related genes *TRIM25* and *NR6A1* (*p* = 0.0098 and *p* = 0.0352) (Figure 5). Among apoptotic genes, this concentration of BPA upregulated the expression of *BID*, *IKBKG*, and *PPID* (*p* = 0.0410, *p* = 0.0412, *p* = 0.0370, respectively) and downregulated the expression of *CASP3* (*p* = 0.0134)*,* as shown in Figure 6.

As expected, the highest concentration of BPA (100 µM) had pronounced effects on gene expression in GCs. It upregulated the expression of the steroidogenesis-related genes *GPER30* and *AR* (*p* = 0.0151 and *p* = 0.0016) and downregulated the expression of *FOXO1* and *UGT2B15* (*p* = 0.0025 and *p* = 0.0031)*,* which are also associated with steroidogenesis (Figure 5). This concentration of BPA also upregulated the expressions of the apoptosis-related genes *BID*, *PPID*, and *IKBKG* (*p* = 0.0042, *p* = 0.0062, and *p* = 0.0053, respectively), as shown in Figure 6. We also observed a trend toward upregulated expression of the apoptotic gene *CASP3* after exposure of GCs to a 100 µM concentration of BPA; however, the change did not reach statistical significance (*p* = 0.0717). These results suggest that BPA modifies the expression of numerous apoptosis and steroidogenesis-related genes in GCs exposed to both lower environmentally relevant concentrations and the highest toxicological concentration of BPA.

### 2.5. Functional Annotation of Upregulated Genes in GCs Exposed to Different Concentrations of BPA

The gene most sensitive to the action of BPA was the apoptotic gene *PPID1*. Compared with the control, the expression of this gene was significant at all three concentrations of BPA for 72 h (Table 1). This gene is proposed to act as a co-chaperone in unligated steroid receptor heterocomplexes, has the potential to exert tissue-specific receptor activity control, and has a preference for estrogen receptor complexes [19]. It is involved in cytoplasmic dynein-dependent movement of the receptor from the cytoplasm to the nucleus and is linked to the cellular apoptosis pathways, where it plays a critical role in the survival of senescent cells [20], increases oxidative stress, and causes cell injury and apoptosis [21].

Furthermore, there were three genes, steroidogenesis-related *AR* and apoptotic *BID* and *IKBKG,* whose expression was upregulated in GCs exposed to higher concentrations of BPA (0.1 µM and 100 µM), as shown in Table 1. *AR* is the androgen receptor gene. Its encoded protein functions as a steroid-hormone-activated transcription factor. ARs are nuclear receptors that include progesterone receptors. *BID* induces caspases and *IKBKG* encodes the regulatory subunit of the inhibitor of kappa-B kinase (IKK) complex, which activates NF-kappa-B, resulting in activation of genes involved in cell survival and other pathways [19].

We also identified three steroidogenesis-related genes that were upregulated in GCs exposed to specific BPA concentrations: *NR6A1* (0.001 µM), *HSD3B1* (0.1 µM), and *GPER30* (100 µM) (Table 1). *NR6A1* encodes an orphan nuclear receptor that is a member of the nuclear hormone receptor family and may be involved in germ cell development [19]. *GPER30* encodes the G-protein-coupled estrogen receptor that binds to 17-beta-estradiol (E2) with high affinity, affecting numerous intracellular signaling pathways [19]. Unlike these two genes, *HSD3B1* encodes an enzyme that catalyzes the oxidative conversion of delta-5-3-beta-hydroxysteroid precursors into delta-4-ketosteroids, which is required for the production of all classes of steroid hormones [19].

### 2.6. Functional Annotation of Downregulated Genes in GCs Exposed to Different Concentrations of BPA

Among the downregulated genes were two steroidogenesis-related genes, *TRIM25* and *UGT2B15*, which were downregulated in GCs exposed to the environmentally relevant concentrations of BPA, and one gene, *FOXO1*, which was downregulated in cells exposed to the highest concentration of BPA (100 µM). *TRIM25* is known to mediate estrogen action [22], and *UGT2B15* plays a role in the regulation of estrogen and androgen [19]. *FOXO1* belongs to the forkhead family of transcription factors [19]. It may be involved in the regulation of the expression of genes involved in the production of gonadotropins, and mice conditional knockouts of *FOXO1* have demonstrated that *FOXO1* plays an important role in ovarian granulosa cell proliferation and apoptosis [23]. In addition to genes associated with steroidogenesis, exposure of GCs to environmentally relevant concentrations of BPA (0.001 or 0.1 µM) inhibited the expression of two apoptotic genes, *RPS6KA3* and *CASP3*. *RPS6KA3* encodes a member of the RSK (ribosomal S6 kinase) family of serine/threonine kinases that regulate diverse cellular processes such as cellular growth, motility, survival, and proliferation [19]. Finally, the apoptotic gene *CASP3* encodes the protease that acts as a major effector caspase involved in the execution phase of apoptosis by catalyzing the cleavage of many proteins [24,25,26,27,28,29].

Table S1 contains a list of genes, specifically involved in apoptosis or steroidogenesis, that were significantly altered in GCs after exposure to BPA and their corresponding protein names. The table also includes a description of their main general functions based on information from the Human Protein Atlas (https://www.proteinatlas.org/, https://www.ncbi.nlm.nih.gov/gene (NCBI Gene Database), accessed on 22 November 2024).

### 2.7. Protein Expression

**Figure 7:**
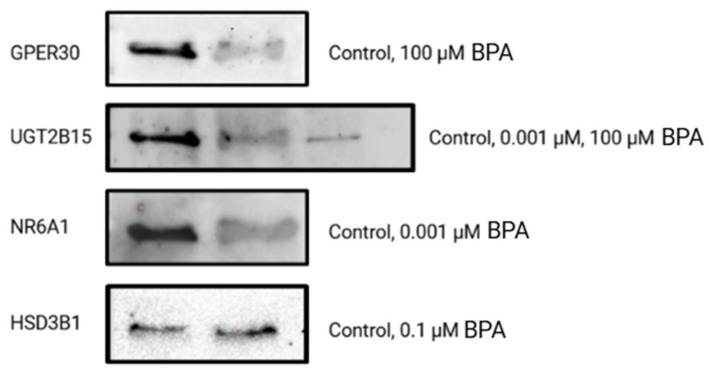
Western blot analysis of control and BPA-exposed granulosa cells for five selected proteins. The analysis was performed to confirm the expression of steroidogenesis and apoptosis-related proteins. The results show that the proteins GPER30, UGT2B15, NR6A1, and GSD3B1 were expressed in control and granulosa cells exposed to different concentrations of BPA, including environmentally relevant ones. However, the protein BID was not expressed in granulosa cells at all.

To confirm the molecular genetic data for five genes that were significantly differentially expressed in GCs after 72 h of exposure to BPA concentrations of 0.001, 0.1, and 100 µM, the expression of five selected proteins (GPER30, HSD3B1, NR6A1, UGT2B15, and BID) was analyzed in GCs using Western blot analysis relative to the total protein count. This analysis confirmed the expression of four proteins, GPER30, HSD3B1, NR6A1, and UGT2B15, while protein BID was not expressed in GCs at all (Figure 7). No target protein was expressed in the negative control or in the serum.

**Figure 8:**
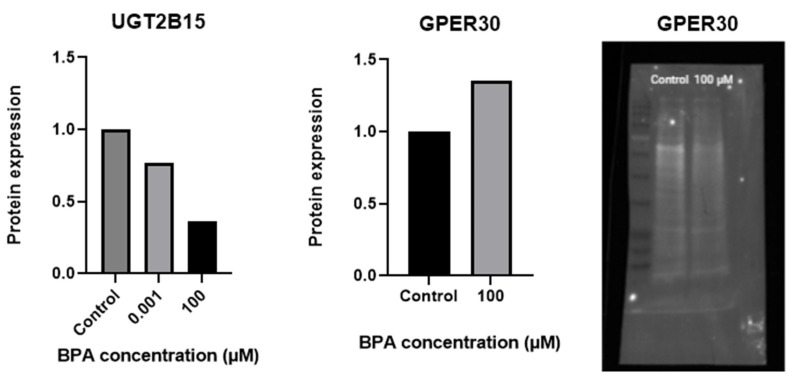
Fold changes in the expressions of the steroidogenesis-related proteins GPER30 and UGT2B15, normalized to total protein in granulosa cells exposed to a 100 µM concentration of BPA in comparison with a non-exposed control. Exposure of granulosa cells to 100 µM BPA increased the expression of GPER30 and decreased the expression of UGT2B15, similar to the corresponding gene expression; however, these differences were relatively small. The total protein blot for GPER30 is included to show overall protein loading across the wells. The 100 µM BPA-treated group shows visibly lower total protein due to a significantly reduced cell count under this condition. This shows that, despite a visually weaker band, normalization reveals a relative increase in GPER30 expression in the 100 µM BPA group.

Similar to gene expression, exposure of GCs to a 100 µM concentration of BPA increased the expression of GPER30 and decreased the expression of UGT2B15, both associated with steroidogenesis (Figure 8). However, the differences were relatively small (1.35-fold change at 100 µM BPA for GPER30, 0.77-fold change at 0.001 µM BPA, and 0.36-fold change at 100 µM BPA for UGT2B15) compared with the control. We could not confirm the quantitative differences for the other proteins (Figure S3), mainly due to a limited amount of material available for analysis. Uncropped membranes and no-stain labelling pictures are depicted in Figure S2.

## 3. Discussion

In the present study, we examined the effects of BPA on human GCs in vitro, focusing on its impact at both environmentally relevant (0.001 and 0.1 µM) and toxicological (100 µM) concentrations after two exposure times (24 and 72 h). We studied how BPA affects GC viability, counts, steroid hormone production, and expression of genes and proteins involved in steroidogenesis and apoptosis. No significant changes were observed in the studied parameters after 24 h of exposure; however, after 72 h, the toxicological concentration of BPA significantly reduced GC viability and counts and lowered the production of estradiol and progesterone, with lower concentrations in spent culture medium. Moreover, BPA significantly affected the expression of genes associated with steroidogenesis and apoptosis, mostly demonstrated for the first time in this context, at all tested BPA concentrations, including environmentally relevant ones.

As expected, exposure to 100 µM BPA drastically reduced the viability and total cell count of GCs compared with the control group. These findings are consistent with multiple studies on GCs that have reported a significant time-dependent decrease in cell viability, particularly at higher toxicological concentrations of BPA (100 µM and above) [12,30,31,32,33,34,35]. Reduced cell viability, observed after exposure to 100 µM BPA, aligns with the significantly upregulated apoptotic gene expression found under this condition. Upregulated levels of *BID*, *PPID*, and *IKBKG* expression suggest that high concentrations of BPA induce apoptosis through the intrinsic apoptotic pathway, primarily by promoting mitochondrial outer membrane permeabilization, resulting in the release of cytochrome C in the cytosol [36,37,38]. These observations support the findings of other studies, focused on BPA and GCs, demonstrating that BPA triggers cellular apoptosis by disrupting metabolism and homeostasis, leading to oxidative stress and mitochondrial dysfunction. Interestingly, we also observed significant upregulation of the cell survival-related gene *IKBKG* and concurrent downregulation of *FOXO1*, which is also essential for the transcription of apoptotic genes.

The highest concentration of BPA, 100 µM, also caused significant decreases in estradiol and progesterone biosynthesis, measured as the concentrations of these hormones in spent culture medium. For progesterone, this was found even after normalization to 1000 viable cells, while estradiol remained unaffected after normalization. These results align with the majority of previously published studies demonstrating that BPA disrupts and impairs steroid hormone synthesis in multiple GC models at toxicological concentrations [31,34,35,39,40,41,42,43,44]. Interestingly, Lebachelier de la Riviere and colleagues found results similar to ours, noting that 50 µM BPA caused a decrease in progesterone but not in estradiol concentration, measured in spent culture medium [45].

While decreased steroid hormone production is typically expected to be associated with decreased mRNA levels of key steroidogenic genes encoding enzymes that are involved in steroidogenesis [32,46], in our study, none of the genes involved in progesterone synthesis (*StAR*, *CYP11A1*, *HSD3B1/2*) or regulation (*PGR*, *PELP1*, *NCOs*) were impacted by 100 μM BPA. Similar findings have been reported in other studies, which, despite observing a significant decrease in progesterone levels in the culture medium, did not detect negative changes in the expression of genes directly involved in progesterone biosynthesis. Bujnakova Mlynarcikova et al. reported that exposure to 100 µM BPA for 72 h drastically reduced progesterone levels in porcine GCs but did not affect the expression levels of *StAR*, *CYP11A1*, *HSD3B*, or *CYP19A1* [47]. Zhou et al. and Qi et al. reported a significant decrease in progesterone levels at 10 μM BPA, accompanied by an increase in *StAR* mRNA, with no changes in *CYP11A1* and *HSD3B2* [31,48]. Meanwhile, Samardzija et al. demonstrated similar results at 50 and 100 μM BPA, which significantly decreased progesterone production in immature rat GCs but increased *StAR*, *CYP11A1,* and *HSD3B1*. These consistent results suggest that BPA can affect progesterone production in GCs independently of alterations in the expression of steroidogenic enzymes. Some studies suggest that the decrease in progesterone production could be due to altered expression of genes involved in cholesterol transport, specifically *ABCA1* and *SREBP-1* [31,41], or progesterone-regulating genes such as *FDX* and *FDXR* [35]. While we did not directly examine cholesterol transport or specific progesterone-regulating genes, *SREBP-1* was included in our analysis but did not show significant alteration. Our findings indicate that BPA may interfere with steroidogenesis through alternative mechanisms, particularly through altered expression of hormone receptors. An elevated expression of *GPER30*, *G protein-coupled estrogen receptor 30*, in GCs after exposure to 100 µM BPA could explain the impaired progesterone synthesis in our study, as it can reduce the activity of steroidogenic enzymes and divert estrogen signaling [49]. We also observed an increase in the expression of *AR*, which plays a pivotal role in mediating the effects of androgen on follicular survival and development and could, if overexpressed, contribute to disrupted steroidogenesis and impaired proliferation of follicular cells [50,51].

Above all, the effects of environmentally relevant concentrations of BPA found in the environment and in biological samples collected from women are important. According to our results, environmentally relevant concentrations of BPA (0.001 and 0.1 µM) appear to compromise both steroidogenic and apoptotic gene expressions. Some of the affected genes are the same as those altered at the highest concentration of BPA (100 µM), but with less statistical significance compared with this concentration. Increased *AR* and *HSD3B1* gene expression in GCs following exposure to 0.1 µM BPA highlights BPA’s endocrine-disrupting characteristics even at environmentally relevant doses. It should be noted that the research on BPA’s effects on *AR* expression is limited, and the results in the literature are inconsistent. In some studies, BPA downregulated *AR* expression in ovine GCs, while in other studies (such as ours), it upregulated *AR* expression in human GCs [12,39,52]. Consequences of upregulated *AR* expression in human granulosa and theca cells are strongly linked to hyperandrogenism and polycystic ovary syndrome (PCOS), as women with PCOS frequently exhibit elevated *AR* levels and androgen activity [53,54,55].

Regarding *HSD3B1*, BPA has previously been shown to affect its expression at environmentally relevant and toxicological concentrations, with variations depending on the GC model [40,41,42]. While elevated *HSD3B1* mRNA levels are typically associated with increased steroidogenesis, this was not observed in our study, based on measuring hormone concentrations in spent medium. Neither protein expression levels nor actual steroid hormone concentrations showed a notable increase. Similar findings were reported in a study on murine GCs, where higher levels of *HSD3B1* expression were not followed by increased progesterone concentration in the spent medium [41]. This discrepancy might be associated with the simultaneous upregulation of pro-apoptotic genes such as *BID*, *IKBKG*, and *PPID*, suggesting that cellular homeostasis is disrupted even at these concentrations.

Interestingly, the effects of the lowest concentration of BPA, 0.001 µM, reflecting the average follicular fluid BPA levels in women [14,15,16,17,18], revealed significant changes in gene expression. Alongside the upregulation of the stress and apoptosis-related gene *PPID* and the steroidogenesis-related gene *NR6A1*, BPA caused notable downregulation of the steroidogenesis-related genes *TRIM25* and *UGT2B15*, and the apoptosis-related genes *RPS6KA3* and *CASP3*. This indicates that steroidogenesis in GCs is impaired, possibly due to alterations in *NR6A1* expression associated with an orphan hormone nuclear receptor involved in germ cell development [56], and *TRIM25* and *UGT2B15* expression, regulated by estrogen and androgen actions [22,57,58]. The apoptotic gene *CASP3* was mostly downregulated due to possible anti-apoptotic mechanisms, including one of the key cell survival factors—PPID [59,60]. GCs exposed to higher concentrations of BPA (0.1 and 100 µM) showed significantly upregulated expression of *PPID* and other apoptotic genes such as *BID* and *IKBKG*. In spite of the upregulated expression of the apoptotic gene *BID*, there was no expression of the corresponding protein in GCs. It is possible that GCs stored the *BID* mRNA without further translation, a phenomenon that can occur in other types of cells [61].

Changes in *PPID*, *TRIM25*, *RPS6KA3*, and *CASP3* expression after exposure to 0.001 µM BPA also suggest a cellular response to oxidative and mitochondrial stress [62,63,64,65]. Such results indicate that BPA in GCs induces cellular stress and a consequent stress response, even at environmentally relevant concentrations of BPA, and support other published data on environmental BPA concentrations [12,66,67,68]. Additionally, BPA shows the ability to interfere with growth and immune signaling pathways, ultimately disrupting cellular homeostasis. The varying effects of different BPA concentrations on gene expression align with findings that BPA often exhibits a non-monotonic dose–response pattern [1,69,70]. Low, environmentally relevant concentrations of BPA can have significant, albeit not necessarily toxic, biological impacts on GCs due to BPA’s endocrine-disrupting characteristics.

## 4. Materials and Methods

### 4.1. Sample Collection

This study was approved by the National Medical Ethics Committee of the Republic of Slovenia (No. 0120-448/2020-3). Waste follicular fluid was collected after oocyte retrieval from three patients undergoing a process of in vitro fertilization after a previous short antagonist protocol of hormonal ovarian stimulation. Written informed consent was provided by each participating woman. Each patient received daily subcutaneous injections of 225 IU of recombinant follicle-stimulating hormone (Gonal-F; Merck KGaA, Darmstadt, Germany) from the second day of their menstrual cycle. Premature luteinizing hormone surge was prevented using a gonadotropin-releasing hormone antagonist (Cetrotide; Merck KGaA, Darmstadt, Germany) from day 7 of the menstrual cycle. When at least 3 follicles reached 18 mm or more in diameter, oocyte maturation was triggered using recombinant human chorionic gonadotropin (Ovitrelle; Merck KGaA, Darmstadt, Germany). Oocyte retrieval was scheduled approximately 36 h after triggering injection. All three patients were 35 years or younger, were treated because of male-factor infertility, had at least 6 oocytes retrieved, and had a body mass index of 30 or less.

### 4.2. Isolation of Human Granulosa Cells from Follicular Fluid

During the oocyte retrieval procedure, follicular fluid was aspirated using an ultrasound-guided transvaginal aspiration system and collected in test tubes. Oocytes were identified and isolated under a microscope, and waste follicular fluid containing granulosa cells was used for further analysis. The waste follicular fluid was transferred into centrifuge tubes and centrifuged at 1000× *g* for 3 min at 21 °C. After this, follicular fluid-derived cells were separated from blood cells via density gradient centrifugation [71,72,73,74,75]. The pellet was resuspended in phosphate-buffered saline (PBS) (Gibco, Waltham, MA, USA) and layered onto a 50% sterile colloid/isotonic salt solution (PureSperm, Nidacon, Gothenburg, Sweden) and centrifuged at 400× *g* for 30 min at 21 °C [74]. After centrifugation, the follicular cells with predominant GCs were removed from the ring-like interface level (Figure S1), resuspended in PBS, and centrifuged at 400× *g* for 10 min. The pellet was resuspended in fresh and sterile DMEM-F12 medium (Gibco, Waltham, MA, USA) and seeded in a 12-well plate.

### 4.3. Cell Culture

Granulosa cells (GCs) from each patient were seeded into four 12-well plates containing fresh DMEM-F12 medium supplemented with 10% fetal bovine serum (FBS) (Gibco, Waltham, MA, USA) and 1% penicillin–streptomycin solution (Gibco, Waltham, MA, USA). The cells were cultured at 37 °C in an atmosphere of 6% CO_2_ for 24 h prior to bisphenol A (BPA (Sigma Aldrich, St. Louis, MO, USA) treatment. Following this initial incubation, two 12-well plates were designated for 24 h exposure and two for 72 h exposure. Of these, one plate from each time point was allocated for gene expression analysis, and the other for Western blot sampling. Within each 12-well plate, three wells per column contained triplicates of cells under identical conditions: the first column served as the control group, the second column for 0.001 µM BPA, the third column for 0.1 µM BPA, and the fourth column for 100 µM BPA.

### 4.4. Cell Viability and Counts

Cells were seeded into 12-well plates and treated in the presence or absence of three different concentrations of BPA for 24 or 72 h. After the treatment, the cells were detached from the plates with TrypleS (Gibco, Waltham, MA, USA), centrifuged, and resuspended in FBS. We used the live/dead staining method (Trypan blue method) to assess cell viability and count. In this method, dead cells take up the trypan blue dye and are stained blue, while live cells exclude this dye and are not stained. A 100 µL aliquot of cell suspension was taken from each sample and mixed with an equal volume of 0.4% trypan blue (Gibco, Waltham, MA, USA). The mixture was incubated at room temperature for 2–3 min before assessing total cell count, as well as the number of live (unstained) and dead (blue-stained) cells. Cell viability (percentage of live cells) and total cell count were determined using an automatic cell counter (Corning, New York, NY, USA) equipped with CytoSmart 1.5.0.16380 software (Axion BioSystems, Atlata, GA, USA) based on the trypan blue dye exclusion method. The results for counting and viability measurements were expressed as the number of cells/mL of culture medium and as the percentage of living and dead cells, respectively.

### 4.5. Progesterone and Estradiol Assays in Spent Culture Medium

For steroid hormone assay, the hGCs were cultured in 12-well plates. After 24 or 72 h of treatment with different concentrations of BPA, the exhausted culture medium was collected and assessed using electrochemiluminescence immunoassay (ECLIA, Roche, Basel, Switzerland). A small volume of each sample was mixed with biotin-labeled estradiol/progesterone-specific antibodies and incubated at room temperature for 30 min. Following the first incubation, streptavidin-coated microbeads and a ruthenium complex-labeled estradiol/progesterone derivative were added to each sample and incubated for another 30 min to form antibody–hapten complexes. After the second incubation, the reaction samples were analyzed with an ECLIA analyzer, based on measuring chemiluminescence emission using a photomultiplier. Unbound substances were removed with the ProCell M kit (Roche Diagnostics, Rotkreuz, Switzerland). Concentrations of both hormones were calculated using a calibration curve obtained through specific two-point calibration. Each sample was measured in duplicate.

### 4.6. Real-Time Polymerase Chain Reaction (qPCR) and Gene Expression

For each BPA treatment or control, three biological replicates (in three women) were analyzed. For each biological replicate, cells were taken from three different wells in the culture plate (technical replicates) and pooled into one sample to concentrate the cells.

Total RNA was isolated using the PureLink RNA Mini Kit (Thermo Fisher Scientific, Waltham, MA, USA) following the manufacturer’s protocol. Genomic DNA contamination was removed with the Invitrogen™ PureLink™ DNase Set (Thermo Fisher Scientific, Waltham, MA, USA), followed by measuring the quantity and quality of isolated RNA using Qubit™ RNA High Sensitivity kit, Qubit RNA IQ Assay Kit (both Thermo Fisher Scientific, Waltham, MA, USA). Based on the quantified RNA, samples were normalized and reverse-transcribed into cDNA using the High-Capacity cDNA Reverse Transcription Kit (Thermo Fisher Scientific, Waltham, MA, USA). The reverse transcription protocol included incubation at 25 °C for 10 min, 37 °C for 120 min, and 85 °C for 5 min. The cDNA was mixed with TaqMan™ Fast Advanced Master Mix (Thermo Fisher Scientific, Waltham, MA, USA) and loaded into TaqMan Arrays for the simultaneous analysis of 96 apoptosis-related genes (TaqMan™ Array, Human Cellular Apoptosis Pathway, Fast 96-well, cat. no. 4418762, Applied Biosystems/Thermo Fisher Scientific, Waltham, MA, USA) and 96 steroidogenesis-related genes (TaqMan™ Array, Human Estrogens, Fast 96-well, cat. no. 4418732, Applied Biosystems/Thermo Fisher Scientific, Waltham, MA, USA). The TaqMan^®^ Array Human Cellular Apoptosis Pathway 96-well Plate contains 92 assays for genes associated with the cellular apoptosis pathway, and the TaqMan^®^ Array 96-well Human Estrogens Plate targets genes that code for enzymes involved in the synthesis of estrogens and the estrogen receptors whose expression mediates the effects of estrogens. The panel also includes genes that are targets of estrogen signaling. Several genes associated with other steroid hormones, such as progesterone (*StAR*, *HSD3B1/2*, *CYP11A1*, *PGR*, *CYP17A1*) and testosterone (*HSD3B1/2*, *AR*, *StAR*, *SRD5A2*, *CYP11A1*, *CYP17A1*, *CYP19A1*), are also included in the panel due to their close associations with estrogen synthesis and function. The steroidogenesis- and apoptosis-related genes that were analyzed are presented in List S1. Each array contained pre-dried, lyophilized assays. *GAPDH*, *HPRT1*, and *GUSB* genes were used as internal references on both plates. Amplification was performed on the ViiA™ 7 Real-Time PCR System under the following conditions: initial denaturation at 95 °C for 2 min, followed by 45 cycles of denaturation at 95 °C for 1 s, and annealing and extension at 60 °C for 20 s. Ct values were obtained from the Quant Studio 7 app, and the expression levels of target genes were calculated using the comparative Ct method (ΔΔ Ct method) [76].

Gene expression was first normalized using three housekeeping genes: *GAPDH*, *HPRT1*, and *GUSB*. The Ct (threshold cycle) values of these housekeeping genes were used to adjust the expression of each gene. After this, a second normalization step was performed by subtracting the delta Ct value of the control group from the delta Ct value of the gene.

To calculate fold change for graphical representation, the formula 2^(−ΔΔCt) was used. The ΔCT data were then analyzed using ANOVA with a Dunnett post hoc test, comparing the experimental groups with the control group [76,77].

### 4.7. Western Blot and Protein Expression

Using the Western blot method, we examined encoded protein expression of five selected genes whose expression was affected by exposure of GCs to BPA. The purpose was only to determine whether these genes were also expressed at the protein level. Due to limited biological material, all three biological replicates were combined into one sample. Total proteins from GC samples treated with various concentrations of BPA were extracted using RIPA buffer supplemented with Complete Mini Protease Inhibitor Cocktail (Roche Diagnostics, Rotkreuz, Switzerland). Due to the low cell number, the resulting total protein concentrations were relatively low, preventing us from normalizing the protein levels across all samples. After extraction, total protein concentration was quantified using the Pierce BCA Protein Assay Kit (Thermo Scientific, Waltham, MA, USA). Following quantification, samples were denatured by adding 1X NuPage LDS buffer (Invitrogen, Carlsbad, CA, USA) and 1 µL of 1M DTT, then heated at 95 °C for 5 min. The denatured samples were loaded onto pre-cast Mini-PROTEAN electrophoresis gels (BioRad, Hercules, CA, USA) and subjected to electrophoresis at 120 V. Subsequently, Western transfer was conducted at 100 V for 90 min. After the transfer, the Immobilion-P PVDF membrane (Merck Millipore, Burlington, MA, USA) was incubated in a No-Stain labeling solution (Invitrogen, Carlsbad, CA, USA) to label total proteins, followed by blocking in 5% skimmed milk/TBST. The membrane was then incubated overnight at 4 °C with primary antibodies (Table 2; all sourced from Abcam, Cambridge, UK) and subsequently with secondary antibodies (goat anti-mouse IgG HRP (1:4000, Santa Cruz Biotechnology, sc-2004, Dallas, TX, USA) and goat anti-rabbit IgG-HRP (1:4000, Santa Cruz Biotechnology, sc-2004, Dallas, TX, USA) for 1 h the next day. The membrane was washed three times with TBST for 15 min between and after the incubations. The iBright Imaging System (Thermo Fisher Scientific, Waltham, MA, USA) and SuperSignal™ West Femto Maximum Sensitivity Substrate detection kit (Thermo Scientific, Waltham, MA, USA) were utilized to visualize both total and target protein bands, which were later normalized and quantified using ImageJ 1.54g software (https://imagej.net/ij/download.html). Protein quantification was performed using a non-stain labeling approach, which allowed the normalization of detected protein based on total protein loaded for each sample [78]. The fold change in expression in the sample group was calculated relative to the control group, which was set to 1 [79].

Negative controls were included to assess antibody specificity. These were represented by samples that were processed without the primary antibody, using only the secondary antibody to confirm the absence of non-specific binding. For protein expression controls, serum was used as a sample lacking the target protein to validate the specificity of detection.

### 4.8. Statistical Analysis

Statistical analysis was conducted using GraphPad Prism 9.0.0 software (GraphPad Software, LLC, Boston, MA, USA). For cell count, cell viability, progesterone and estradiol concentrations in spent culture medium, and gene expression, parametric analysis of variance (ANOVA) was performed. This approach was supported by a prior test for normal distribution using the Shapiro–Wilk test. Differences between treatment groups and the control group were determined using Dunnett’s post hoc test.

A *p*-value ≤ 0.05 was considered statistically significant. Statistical significance was represented as follows: *p* ≤ 0.05 (*)*, p* ≤ 0.01 (**), and *p* ≤ 0.0001 (***).

## 5. Conclusions

In conclusion, our findings support the growing evidence that human BPA disrupts steroidogenesis and induces apoptosis in GCs, with significant implications for ovarian function and fertility. Disrupted steroidogenesis and apoptosis were pronounced at the highest concentration of BPA, 100 µM, both in terms of altered gene expression and practical outcomes, such as estradiol and progesterone synthesis, reflected by the concentrations of these hormones in spent culture medium, cell counts, and cell viability. Importantly, the reductions in GC viability and cell counts appear to be primarily driven by increased apoptosis, highlighting its central role in the observed effects. Although environmentally relevant concentrations of BPA showed no significant impact on cell viability or steroidogenesis, they significantly altered the expression of several apoptotic and steroidogenic genes, which were mostly demonstrated for the first time in this context. This suggests that BPA may interfere with important cellular pathways at environmental levels and environmentally relevant concentrations, potentially leading to long-term disruptions in hormonal receptors and regulation of hormonal action and cellular homeostasis. These results emphasize the need for further research into the long-term effects of BPA exposure on reproductive health, particularly at relatively low and environmentally relevant concentrations that are commonly encountered in everyday environments and human biological samples.

## Figures and Tables

**Table 1:** Genes whose expression was significantly up- or downregulated in granulosa cells after 72 h exposure to different BPA concentrations. The steroidogenesis-related genes that were altered include *NR6A1* (upregulated at 0.001 µM BPA), *TRIM25* (downregulated at 0.001 and 0.1 µM BPA), *UGT2B15* (downregulated at 0.001 and 100 µM BPA), *HSD3B1* (upregulated at 0.1 µM BPA), *AR* (upregulated at 0.1 and 100 µM BPA), *GPER30* (upregulated at 100 µM BPA), and *FOXO1* (downregulated at 100 µM BPA). The genes involved in apoptosis include *PPID* (upregulated at all BPA concentrations), *CASP3* (downregulated at 0.001 and 0.1 µM BPA), *RPS6KA3* (downregulated at 0.001 µM BPA), *BID* (upregulated at 0.1 and 100 µM BPA), and *IKBKG* (upregulated at 0.1 and 100 µM BPA).

Genes	Concentrations of BPA		
	0.001 µM	0.1 µM	100 µM
Steroidogenesis			
*NR6A1* *(Nuclear Receptor Subfamily 6 Group A Member 1)*	↑		
*TRIM25* *(Tripartite Motif Containing 25)*	↓	↓	
*UGT2B15* *(UDP Glucuronosyltransferase Family 2 Member B15)*	↓		↓
*HSD3B1* *(Hydroxy-Delta-5-Steroid Dehydrogenase, 3 Beta- And Steroid Delta-Isomerase 1)*		↑	
*AR* *(Androgen Receptor)*		↑	↑
*GPER30* *(G Protein-Coupled Estrogen Receptor 30)*			↑
*FOXO1* *(Forkhead Box O1)*			↓
Apoptosis			
*PPID* *(Peptidylprolyl Isomerase D)*	↑	↑	↑
*CASP3* *(Caspase 3)*	↓	↓	
*RPS6KA3* *(Ribosomal Protein S6 Kinase A3)*	↓		
*BID* *(BH3 Interacting Domain Death Agonist)*		↑	↑
*IKBKG* *(Inhibitor of Nuclear Factor Kappa B Kinase Regulatory Subunit Gamma)*		↑	↑

Legend: ↑ increased expression and ↓ decreased expression.

**Table 2:** List of antibodies used for Western blot analysis.

Antibody	Company	Mono/Polyclonal	Source	Dilution for WB
Anti-RTR antibody	Abcam, Cambridge, UK	Polyclonal	Rabbit	1:250
Anti-G-protein coupled receptor 30 antibody	Abcam, Cambridge, UK	Monoclonal	Rabbit	1:500
Anti-UGT2B15 antibody	Abcam, Cambridge, UK	Monoclonal	Rabbit	1:1000
Anti-Bid antibody	Abcam, Cambridge, UK	Monoclonal	Rabbit	1:2500
Anti-HSD3B1 antibody	Abcam, Cambridge, UK	Monoclonal	Mouse	1:2000

## Data Availability

The datasets generated during the current study are available from the corresponding author upon reasonable request.

## Supplementary Materials

The following supporting information can be downloaded at https://www.mdpi.com/article/10.3390/ijms26094081/s1.
