# Supplementary material for: Bisphenol a Disrupts Steroidogenesis and Induces Apoptosis in Human Granulosa Cells Cultured In Vitro
Source: Int J Mol Sci. 2025 Apr 25;26(9):4081. doi: 10.3390/ijms26094081 (PMC12071243; doi:10.3390/ijms26094081)
Supplement: Supplementary file 1 [file ijms-26-04081-s001.zip › ijms-3584169-supplementary.pdf]

Supplemental material

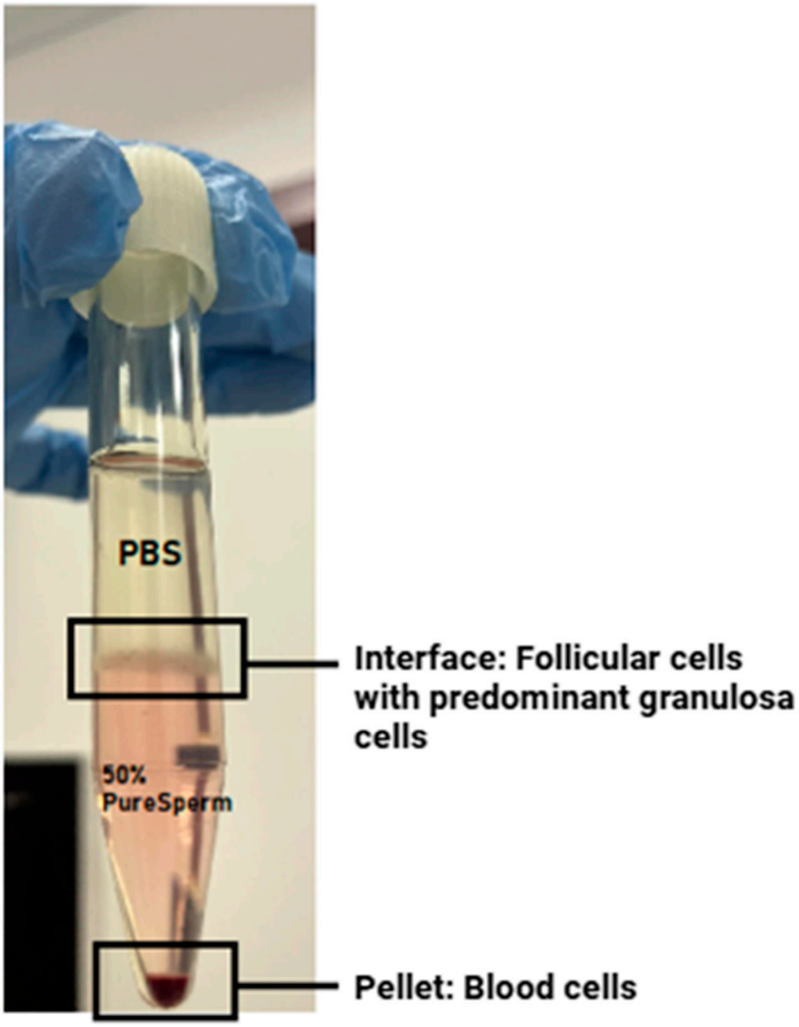

Supplemental Figure 1: After density gradient centrifugation using 50% PureSperm® (Nidacon, Sweden) in a sterile polypropylene centrifuge tube, distinct layers were observed. At the bottom of the tube, a pellet containing blood and immune cells was present, while the phase above consisted of the 50% PureSperm® solution. Between the PureSperm® layer and PBS, a cloudy interface containing follicular cells with predominant (> 80%) granulosa cells was visible. These granulosa cells were carefully removed using a Pasteur pipette and subsequently washed with PBS.

List of genes, included in the Human Estrogens Array plate: *ADCYAP1, BCAR1, BCAR3, CARM1, CEBPB, CYO11CYP19A1, CYP1B1, DDX54, EBAG9, EGF, ERG, ESR1, ESR2M, ESRRA, ESRRG, FGF2, FOXO1, FSHB, FSHR, GABARAPL1, GHRH, GNL3, GNRH1, HMGA1, HMGB1, HMGB2, HSD17B2, HSD17B8, HSPB8, IGF1, IL1A, IL1B, INHA, ISG20, LHCGR, TSKU, MKNK2, MPG, MTA1, NCOA1, NCOA3, NCOA5, NCOA6, NCOA7, NFATC4, NFKB1, NR0B2, NR1I3, NR2C2, NR6A1, NRG1, NRIP1, OVGP1, PELP1, PGR, PHB2, PLG, POURF1, POU4F2, PPARA, PPARGC1A, PPARGC1B, PPID, RBM9, PERG, RLN1, SAFB, SAFB2 SMARCA4, SRD5A2, SREBF1, STS, SULT1E1, TADA3L, TAF10, TCF7, TFF1, TNF, TRIM16, TRIM25, UGT1A8, UGT1A3, UGT1A4, UGT2A1, UGT2B15, UGT2B4, UGT2B7, VIP, NR0B1, GNRH2, NRG3, NRG4, AR, StAR/STARD, HSD3B1, HSD3B2, HSD17B, CYP11A1, CYP17A1, GPER/GP30.*

List of genes, included in the Human Apoptosis Array plate: *AIFM1*, *AKT1*, *APAF1*, *ATM*, *BAD*, *BAK1*, *BAX*, *BBC3*, *BCL2*, *BCL2L1*, *BCL2L11*, *BID*, *BIRC2, CASP3*, *CASP7*, *CASP8*, *CASP9*, CDKN2A, *CFLAR*, *CHEK2*, *CHUK*, *CYCS*, *DAPK3*, *DAXX*, *DFFA*, *DFFB*, *DIABLO*, *EGFR*, *ENDOG*, *F2RL3*, *FADD*, *FAS*, *FASLG*, *HTRA2*, *IGF1*, *IGF1R*, *IKBKB*, *IKBKE*, *IKBKG*, *IL2*, *IL6*, *KDR*, *KIT*, *MDM2*, *MET*, *NFKB1*, *NFKB2*, *NFKBIA*, *NFKBIB*, *NFKBIE*, *P53AIP1*, *PARP1*, *PARP2*, *PARP3*, *PARP4*, *PIK3CA*, *PIK3CB*, *PIK3CD*, *PIK3R1*, *PIK3R2*, *PMAIP1*, *PPID, PRKCA*, *PRKCB*, *PRKCD*, *PRKCE*, *PRKCZ*, *REL*, *RELA*, *RELB*, *RIPK1*, *RPS6KA1*, *RPS6KA2*, *RPS6KA3*, *RPS6KA4*, *RPS6KA5*, *SLC25A4*, *SLC25A5*, *SLC25A6*, *TGFB1*, *TNF*, *TNFRSF10A*, *TNFRSF1A*, *TNFSF10*, *TNFSF12*, *TP53*, *TRADD*, *TRAF2*, *TSPO*, *VDAC1*, *VDAC2*, *VDAC3.*

Additional reference genes: *GAPDH, GUSB, HPRT1*

Supplemental List 1: List of genes, included in the Human Estrogens Array and the Human Apoptosis Array plates.

Official Symbol	Official Full Gene Name	Official Protein name	Process	Function
AR	Androgen receptor	Androgen Receptor	Steroidogenesis	The protein functions as a steroid-hormone activated transcription factor. Upon binding the hormone ligand, the receptor dissociates from accessory proteins, translocates into the nucleus, dimerizes, and then stimulates transcription of androgen responsive genes.
BID	BH3 interacting domain death agonist	BH3 Interacting Domain Death Agonist Protein	Apoptosis	This gene encodes a death agonist that heterodimerizes with either agonist BAX or antagonist BCL2, and thus regulate apoptosis. The encoded protein is a member of the BCL-2 family of cell death regulators. I
CASP3	Caspase 3	Caspase-3	Apoptosis	The protein encoded by this gene is a cysteine-aspartic acid protease that plays a central role in the execution-phase of cell apoptosis. The encoded protein cleaves and inactivates poly(ADP-ribose) polymerase while it cleaves and activates sterol regulatory element binding proteins as well as caspases 6, 7, and 9.
FOXO1	Fork head box 1	Forkhead Box Protein O1	Steroidogenesis	This gene belongs to the forkhead family of transcription factors which are characterized by a distinct forkhead domain. The specific function of this gene has not yet been determined; however, it may play a role in myogenic growth and differentiation.
GPER1	G protein-coupled receptor 1	G Protein-Coupled Estrogen Receptor 1	Steroidogenesis	This gene encodes a multi-pass membrane protein that localizes to the endoplasmic reticulum and a member of the G-protein coupled receptor 1 family. This receptor binds estrogen and activates multiple downstream signaling pathways, leading to stimulation of adenylate cyclase and an increase in cyclic AMP levels, while also promoting intracellular calcium mobilization and synthesis of phosphatidylinositol 3,4,5-trisphosphate in the nucleus. This protein therefore plays a role in the rapid nongenomic signaling events widely observed following stimulation of cells and tissues with estrogen.
HSD3B1	Hydroxy-delta-5-steroid dehydrogenase	3β-Hydroxysteroid Dehydrogenase	Steroidogenesis	The protein encoded by this gene is an enzyme that catalyzes the oxidative conversion of delta-5-3-beta-hydroxysteroid precursors into delta-4-ketosteroids, which leads to the production of all classes of steroid hormones. The encoded protein also catalyzes the interconversion of 3-beta-hydroxy- and 3-keto-5-alpha-androstane steroids.
IKBKG	Inhibitor of nuclear factor kappa B kinase regulatory subunit gamma	NF-kappa-B Essential Modulator	Apoptosis	This gene encodes the regulatory subunit of the inhibitor of kappaB kinase (IKK) complex, which activates NF-kappaB resulting in activation of genes involved in inflammation, immunity, cell survival, and other pathways.
NR6A1	Nuclear receptor subfamily 6 group A member 1	Germ Cell Nuclear Factor	Steroidogenesis	This gene encodes an orphan nuclear receptor which is a member of the nuclear hormone receptor family. Its expression pattern suggests that it may be involved in neurogenesis and germ cell development. The protein can homodimerize and bind DNA, but in vivo targets have not been identified.
PPID	Peptidylprolyl isomerase D	Cyclophilin D	Apoptosis	The protein encoded by this gene is a member of the peptidyl-prolyl cis-trans isomerase (PPlase) family. PPlases catalyze the cis-trans isomerization of proline imidic peptide bonds in oligopeptides and accelerate the folding of proteins. This protein has been shown to possess PPlase activity and, similar to other family members, can bind to the immunosuppressant cyclosporin A.
RPS6KA3	Ribosomal protein S6 kinase A3	Ribosomal Protein S6 Kinase Alpha-3	Apoptosis	This gene encodes a member of the RSK (ribosomal S6 kinase) family of serine/threonine kinases. This kinase contains 2 non-identical kinase catalytic domains and phosphorylates various substrates, including members of the mitogen-activated kinase (MAPK) signalling pathway. The activity of this protein has been implicated in controlling cell growth and differentiation.
TRIM25	Tripartite motif containing 25	E3 Ubiquitin	Steroidogenesis	The protein encoded by this gene is a member of the tripartite motif (TRIM) family. The TRIM motif includes three zinc-binding domains, a RING, a B-box type 1 and a B-box type 2, and a coiled-coil region. The protein is an RNA binding protein, functions as a ubiquitin E3 ligase and is involved in multiple cellular processes, including regulation of antiviral innate immunity.
UGT2B15	UDP glucuronosyltransferase family 2 member B15	UDP-glucuronosyltransferase 2B15	Steroidogenesis	This gene encodes a glycosyltransferase that is invovled in the metabolism and elimination of toxic compounts, both endogenous and of xenobiotic origin. This gene plays a role in the regulation of estrogens and androgens.

Supplemental Table 1 List of genes which were significantly altered in granulosa cells after exposure to BPA and their corresponding protein names, specifically involved in apoptosis or steroidogenesis. The table also includes a description of their main functions based on information from the Human Protein Atlas (https://www.proteinatlas.org/).

GPER30

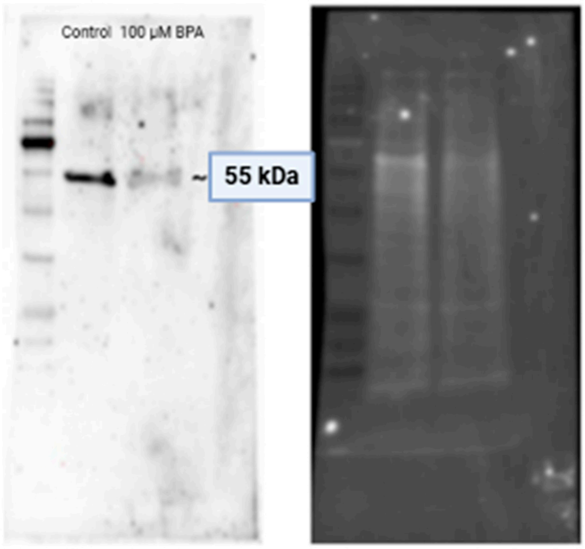

Uncropped original:

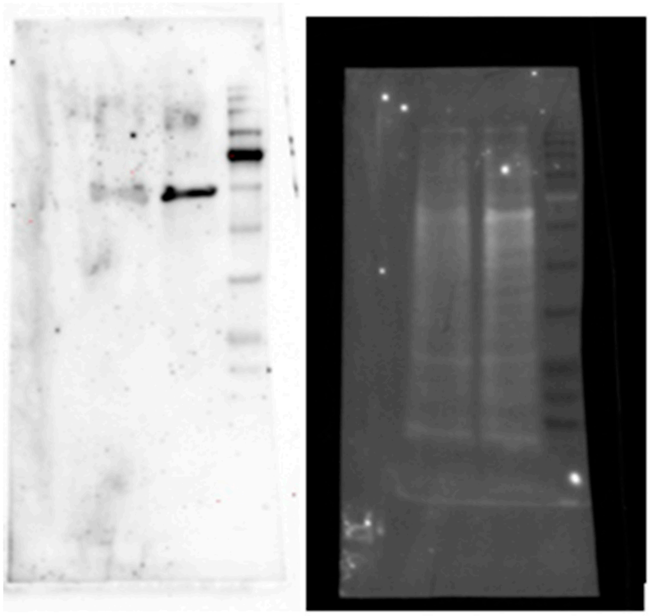

UGT2B15

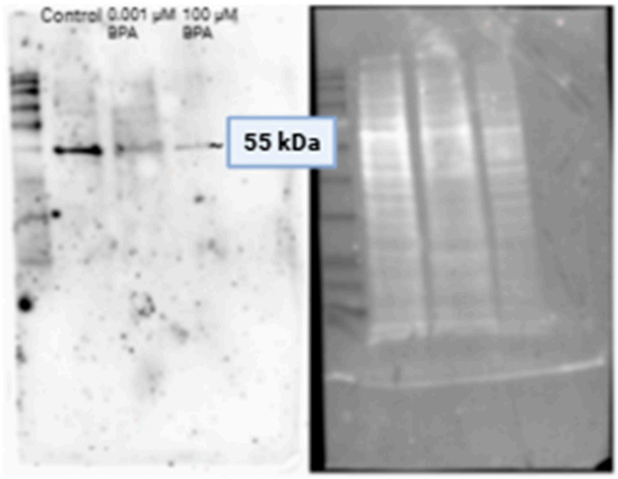

Uncropped original:

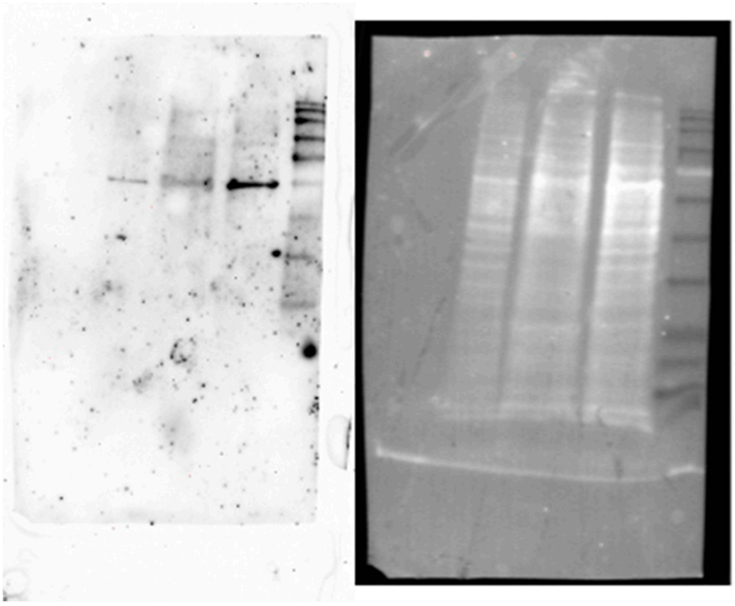

NR6A1 (RTR)

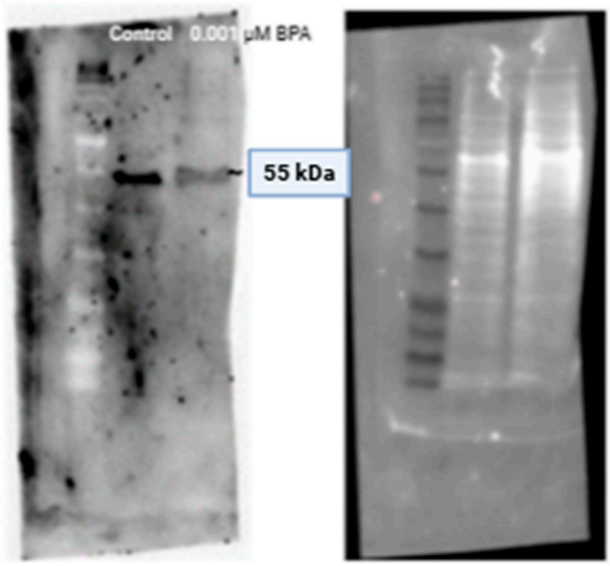

Uncropped original:

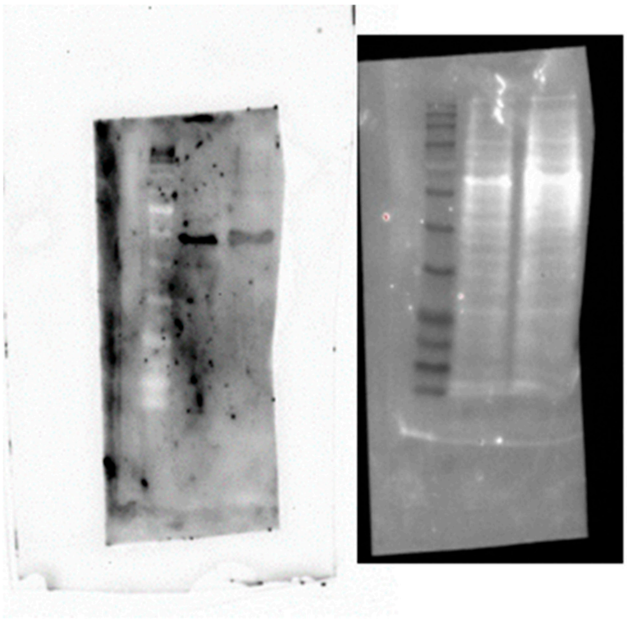

HSD3B1

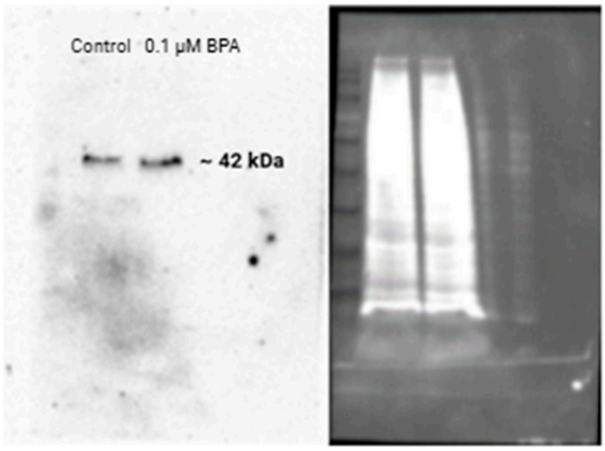

Uncropped original (With/without attached ladder):

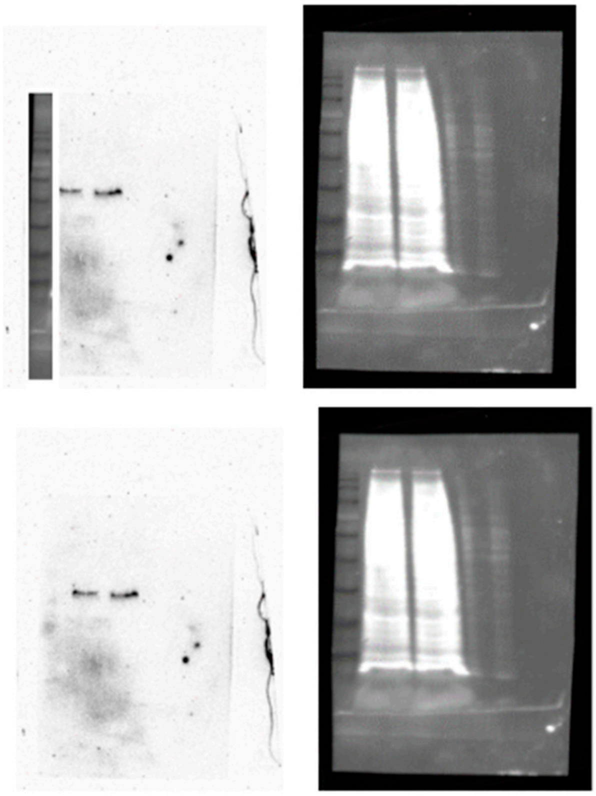

Supplemental Figure 2: Raw Western blot membranes, showing bands and total protein (SuperSignal West Pico Plus channel and Prestained Ladder-Membrane+No-Stain Labeled Membrane channel).

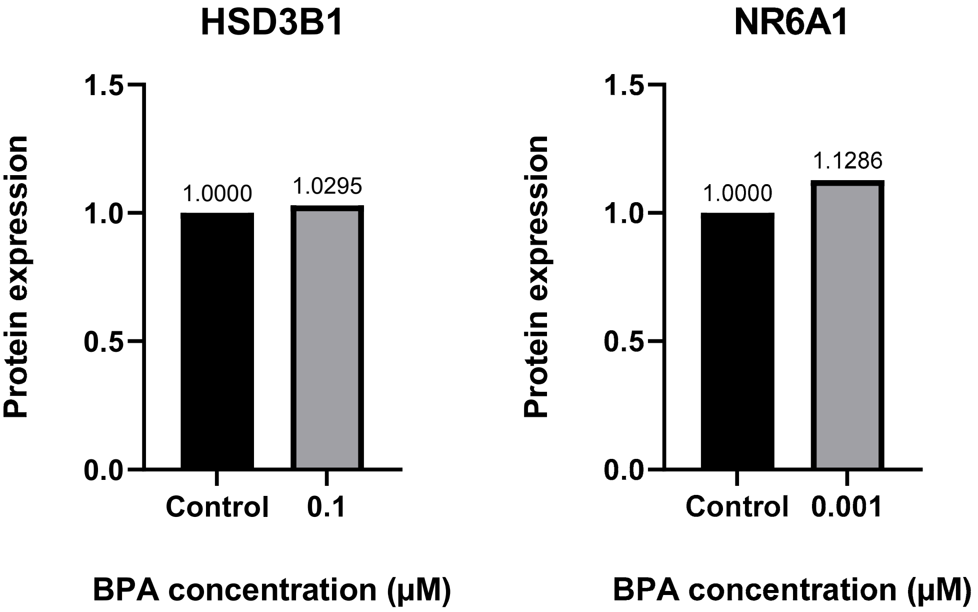

Supplemental Figure 3: Fold changes in expressions of proteins HSD3B1 and NR6A1 as normalized to total protein in granulosa cells exposed to different concentrations of BPA (pictured on graphs) in comparison with non-exposed control.
